# Microbiome/transforming growth factor-β axis as a diagnostic and therapeutic target for MASLD in Egyptian patients

**DOI:** 10.1186/s13099-026-00826-4

**Published:** 2026-04-11

**Authors:** Amani E. Marawan, Omar Ahmed Elmetwally, Shimaa A. Abass, Mohamed M. Marwan, Mohamed M. A. El-Sokkary, Laila A. Eissa

**Affiliations:** 1 Fellow of Bacteriology, Immunology, and Mycology, Faculty of Veterinary Medicine, Shoha Veterinary Teaching Hospital, Mansoura University, Mansoura, 35738 Egypt; 2 Internal medicine department, Hepatology and Gastroenterology unit, Faculty of Medicine, Mansoura University, Mansoura, Egypt; 3 Biochemistry Department, Faculty of Pharmacy, Kafreksheikh University, Kafreksheikh, 33516 Egypt; 4 Department of Pharmacology and Biochemistry, Faculty of Pharmacy, Horus University, New Damietta, 34518 Egypt; 5 Microbiology and Immunology Department, Faculty of Pharmacy, Mansoura University, Mansoura, 35516 Egypt; 6 Department of Biochemistry, Faculty of Pharmacy, Mansoura University, Mansoura, 35516 Egypt; 7 Biochemistry Department, Faculty of Pharmacy, Mansoura University, Mansoura, 35516 Egypt

**Keywords:** MASLD, Gut microbiome, TGF-β, *Bacteroides*, *Bifidobacterium*, Gut–liver axis

## Abstract

Metabolic dysfunction-associated steatotic liver disease (MASLD) has emerged as the leading cause of chronic hepatic disorders worldwide. Gut microbiome dysbiosis influences MASLD pathogenesis *via* the gut–liver axis. This study investigated gut microbiome alterations, biochemical markers, and their diagnostic potential in MASLD. A case control study included 60 Egyptian adults (30 MASLD patients, 30 healthy controls) who underwent comprehensive biochemical profiling and quantitative real-time polymerase chain reaction (qRT-PCR) analysis of selected gut microbiota from fecal samples. Antioxidant biomarkers, including superoxide dismutase (SOD) and malondialdehyde (MDA), were measured spectrophotometrically, while mRNA expression of nuclear factor erythroid 2-related factor 2 (NRF2), transforming growth factor-beta (TGF-β), and signal transducer and activator of transcription 3 (STAT3) was assessed by reverse transcription PCR (RT-PCR). The fibrosis index (FIB-4) was calculated, and diagnostic accuracy was evaluated using receiver operating characteristic (ROC) curves. MASLD patients displayed marked microbiome dysbiosis, with significant enrichment of *Bacteroides* (*p* < 0.001) and depletion of *Bifidobacterium* (*p* < 0.005). TGF-β expression was significantly elevated (*p* < 0.001) and demonstrated excellent diagnostic performance (area under the curve [AUC] = 0.956; sensitivity = 100%; specificity = 83.3%). MDA, FIB-4, and NRF2 showed moderate accuracy, while Bacteroides (AUC = 0.744) and Bifidobacterium (AUC = 0.683) also provided discriminatory value. *Bacteroides* correlated positively with total cholesterol, whereas *Bifidobacterium* correlated inversely. Collectively, elevated TGF-β, increased *Bacteroides*, and reduced *Bifidobacterium* highlight promising diagnostic and therapeutic targets in MASLD. These findings emphasize the mechanistic role of the gut–liver axis and support microbiome-informed roles for early detection and management.

## Introduction

Metabolic dysfunction-associated steatotic liver disease (MASLD) is one of the most prevalent hepatic disorders globally and is currently the chief cause for liver transplantation [1]. Due to the high rise in the worldwide aging population and metabolic risk factors, the burden of MASLD-associated advanced hepatic condition is predicted to double by 2030 [2]. MASLD is characterized by the existence of steatosis in liver tissues, confirmed by imaging or histological assessment, in individuals having concomitant cardiometabolic risk factors, involving hypertension, elevated body mass index, insulin resistance, or dyslipidemia, in the absence of previous alcohol administration [3]. Type 2 diabetes, obesity, metabolic syndrome, and hyperlipidemia represent common comorbidities associated with MASLD. Around 20–30% of individuals with MASLD develop to the more advanced type, metabolic dysfunction-associated steatohepatitis (MASH), which can progress to liver cirrhosis and its associated complications like hepatocellular carcinoma. Globally, the prevalence of liver fibrosis associated with MASLD is supposed to rise by approximately two- to three-fold over the next decade [4].

Importantly, the growing prevalence of metabolic risk factors among adolescents and children serves as a significant threat to global health in the future. In Egypt, rapid socioeconomic transitions have driven notable dietary changes, characterized by greater consumption of sugar-sweetened beverages and calorie-dense snacks, poor in nutrients [5]. Various scoring systems and non-invasive serological markers have been established over the past decade; nevertheless, no one has yet been able to replace liver biopsy fully [6]. Further study is critical to understanding the variability and reliability of novel biomarkers in diagnosing MASLD.

Several studies have revealed a strong relationship between the liver and the gut. This relation regulates the hepatic gut health state and disease development, and it also influences signaling between the host and the gut [7]. The gut microbiomes belong to a large, complicated ecosystem, comprising various microorganisms within the host’s digestive tract. It is estimated that over 10^14^ microbial cells reside in the gut, more than the body’s own cells by a factor of ten [8]. dysregulation in the gut microbiome’s abundance affects the production of microbe-produced substances and metabolites that enter the systemic circulation [9]. Additionally, gut microbiota influences lipid absorption by generating metabolites, including bile acids and short-chain fatty acids (SCFAs), thereby contributing to the development of hepatic steatosis [10]. Although the functional capacity, particularly the metabolic output, of the microbiome is identified as the chief cause in the occurrence and progression of disease, significant variability among metabolomics platforms and difficulties in integrating data across studies have limited the ability to identify consistent microbiome-associated metabolic signatures linked to MASLD [9].

To investigate the distinct alterations in gut microbiome associated with MASLD, we conducted a case-control study involving 60 Egyptian individuals, including subjects with or without MASLD, all with well-defined clinical profiles. Our research utilized RT-PCR to detect the presence of specific microbial populations, including *Bacteroides*, *Bifidobacterium*, and *Providencia*, and others in patient stool samples. This approach enabled us to assess targeted microbial signatures with greater specificity. By integrating microbial data within a consistent analytical framework, we identified disease-specific microbial signatures potentially involved in either promoting or mitigating MASLD progression.

Several studies have reported that dysregulation in the gut microbiota composition has been linked to changes in host biomarkers related to oxidative stress, inflammation, and liver fibrosis [11]. Microbial metabolites and endotoxins can trigger hepatic immune responses and oxidative damage, contributing to the progression of steatosis and fibrosis [12]. Accordingly, our study explored the associations between specific gut microbiomes and some circulating markers such as superoxide dismutase (SOD), malondialdehyde (MDA), nuclear factor erythroid 2–2-related factor 2 (NRF2), transforming growth factor-beta (TGF-β), and fibrosis indices like FIB-4 in MASLD patients. These correlations suggest that gut microbiomes exhibited a mechanistic role in managing the biochemical environment of the host, thereby influencing disease progression.

## Materials and methods

### Sample size calculation

**Hypothesis:Null hypothesis**: No statistically significant difference in inflammatory markers and microbiota distribution in MASLD patients vs. healthy controls.

**Alternative hypothesis:**A statistically significant difference in inflammatory markers and microbiota distribution in MASLD patients vs. healthy controls with large effect size (Cohen’s d = 0.8). This hypothesis was based on previous studies [13].

**Sample size**:

Sample size was calculated by using G*Power software for Windows (version 3.1.9.7). Group sample sizes of 30 per group achieve 86.14% power to reject the null hypothesis of zero effect size when the population effect size is 0.80, and the significance level (α) is 0.050 using a two-sided two-sample equal-variance t-test.

### Study design and participants

This case-control study was performed on 60 participants from the Internal Medicine Department and Gastroenterology Department, Mansoura University Hospital, Mansoura, Egypt, collaborating with the Biochemistry Department, Faculty of Pharmacy, Mansoura University, Mansoura, Egypt. This study was performed from March to September 2024. All participants are sex and age-matched and divided into two groups: The control healthy group, 30 participants from the hospital staff with normal liver function tests, negative hepatitis markers, and unremarkable abdominal ultrasound findings. The MASLD group consists of 30 patients identified with Metabolic Dysfunction-Associated Steatotic Liver Disease (MASLD) according to clinical, laboratory, and imaging criteria. We exclude all subjects with significant alcohol intake, other chronic liver diseases, malignancy, recent use of antibiotics or probiotics, gastrointestinal surgery, inflammatory bowel disease, and subjects under 18 years old, pregnant, or lactation. Detailed medical histories were collected from all participants, followed by thorough clinical examinations, as well as radiological and laboratory evaluations. The protocol of this study was approved by the Ethical Committee of the Faculty of Medicine, Mansoura University (Approval Code: R.23.06.2237) and performed following the principles of the Declaration of Helsinki. Informed written consent was gotten from all participants before enrollment.

### Blood samples and biochemical assays

Under sterile conditions, 10 ml blood specimens were gathered from each participant to conduct the routine laboratory tests. These included liver function tests (alanine aminotransferase (ALT), aspartate aminotransferase (AST), albumin, and total bilirubin), Serum kidney function **(**serum creatinine and uric acid), complete blood counts using Sysmex, Serum lipid profile (total cholesterol and triglyceride), TSH, and international normalized ratio (INR). Fibrosis-4 index (FIB-4) was detected as previously outlined [14].

### Real-time PCR test for detecting the relative expression of gut microbiomes

The real-time quantitative PCR technique was conducted to ascertain the relative expression of gut microbiomes, including *Bacteroides* spp., *Porphyromonas gingivalis*,* Bifidobacteria* spp., *Enterococcus Faecium*, *Fusobacteria* spp., *Pseudomonas aeruginosa*,* Providencia* spp., and *Clostridium* spp., (Table 1). During medical examination, fresh stool samples were obtained from all applicants and instantly kept at − 80 °C. The QIAamp Fast DNA Stool Mini Kit (50) (LOT 163047148) was used for extracting microbial DNA, following the manufacturer’s guidelines. A Nano Drop device (OPTIZEN NanoQ, Mecasys) was used to measure the concentration of DNA spectrophotometrically. Then DNA samples were frozen at −80 °C for further analysis. SYBR Green PCR master mix (Willowfort Co., UK) was used for quantitative DNA measurements from isolated fecal DNA using each forward and reverse primer. The following parameters were adjusted for all real-time reactions: 95 °C for 5 min, followed by 45 cycles of 95 °C for 20 s, an annealing temperature for 20 s (refer to Table 1), and 72 °C for 40 s. The RT-PCR reactions were conducted using the MyGo real-time PCR machine and its associated software. The Ct values were detected and melting curves were analyzed to ensure amplification specificity. 2^−ΔΔCt^ method was used to measure the relative abundance of each microbiome normalized to the total bacterial count in the corresponding specimen.

**Table 1:** The different primers used for detecting different bacterial species

Primer name	Primer sequence		Annealing Tm	Size bp	Citation
*E. Faecium*	F	GCAAGGCTTCTTAGAGA	46.5	564	[15]
	R	CATCGTGTAAGCTAACTTC			
*Bifidobacterium*	F	CTCCTGGAAACGGGTGG	51	551	[16]
	R	GGTGTTCTTCCCGATATCTACA			
*Porphyromonas gingivalis*	F	AATCGTAACGGGCGACACAC	53	594	[17]
	R	GGGTTGCTCCTTCATCACAC			
*Fusobacterium*	F	GGATTTATTGGGCGTAAAGC	51.5	162	[18]
	R	GGCATTCCTACAAATATCTACGAA			
*Providencia*	F	ACCGCATAATCTCTTAGG	43.5	514	[19]
	R	CTACACATGGAATTCTAC			
*Clostridium* sp.	F	CGGTACCTGACTAAGAAGC	50	429	[20]
	R	AGTTTGATTCTTGCGAACG			
*P. aeruginosa*	F	CGAGTACAACATGGCTCTGG	53	116	[21]
	R	ACCGGACGCTCTTTACCATA			
*Bacteroides* sp.	F	AAGGGAGCGTAGATGGATGTTTA	55	193	[22]
	R	CGAGCCTCAATGTCAGTTGC			
*All bacteria*	F	GAGTTTGATCCTGGCTCAG	51	312	[23]
	R	GCTGCCTCCCGTAGGAGT			

### Evaluation of the oxidative stress biomarkers

The concentrations of superoxide dismutase (SOD) and malondialdehyde (MDA) were detected calorimetrically in the serum of all participants based on the previously described method by Nishikimi et al. [24], and Aebi [25], respectively, utilizing available kits (Bio diagnostic, Cairo, Egypt).

### Gene expression analysis of blood biomarkers

The RNA was isolated from frozen blood samples utilizing an available RNA extraction kit (Direct-zol RNA MiniPrep, Zymo Research Co., U.S.A.). Firstly, the triazole reagent was utilized to obtain a high concentration. After that, the obtained supernatant was applied to a mini-spin column. The purity of RNA was detected by the A260/A280 ratio, with an acceptable range of 1.8 to 2.1. One µg of RNA from each sample was used to obtain Complementary DNA according to the instructions of the Quantitect Reverse Transcription kit acquired from Qiagen, Valencia, USA. The primers used are listed in Table 2. Quantitative real-time PCR analysis was conducted utilizing cDNA samples and a Maxima SYBR Green/Fluorescein master mix (Fermentas, United States). 2^−ΔΔ C T^ method was exploited to detect the fold change of the expression of each mRNA corresponding to GAPDH.

**Table 2:** The forward and reverse primer of each gene

Gene name	Primer sequence	Reference
STAT 3	F-5′-GCCAGAGAGCCAGGAGCA3′ R-5′-TGAAGCTGACCCAGGTAGCGCTGC3′	[26]
NRF2	F-5′-CTTTTGGCGCAGACATTCC-3′ R- 5′-AAGACTGGGCTCTCGATGTG-3′	[27]
TGF-β	F-5’-CCCAGCATCTGCAAAGCTC-3’ R- 5’-GTCAATGTACAGCTGCCGCA-3’	[28]
GAPDH	F 5′-TGG TAT CGT GGA AGG ACT CAT-3′ R-5′-ATG CCA GTG AGC TTC CCG TTC AGC-3′	[29]

### Statistical analysis

Data was analyzed using SPSS version 20 (Inc., Chicago, Illinois, USA). Continuous variables were expressed as medians and interquartile ranges (Q1–Q3). The Mann-Whitney test and Brunner-Munzel test (BMT) which is used in medical statistics to compare means between two independent groups involving non-homogeneous group variances, making it an invaluable tool in clinical research [30]. were used to assess differences between the MASLD and control groups. The diagnostic performance of each biomarker and microbiota parameter was evaluated using Receiver Operating Characteristic (ROC) curve analysis, with calculation of AUC (Area Under the Cu rve), sensitivity (SN), specificity (SP), positive predictive value (PPV), negative predictive value (NPV), accuracy (ACC), F1 score, and Matthews’s correlation coefficient (MCC). Correlations between biomarkers, microbiota levels, and lipid parameters (total cholesterol and triglycerides) were assessed using Spearman’s rank correlation coefficient. A p-value < 0.05 was considered statistically significant. Given the multiple correlated biomarkers and microbiota parameters analyzed, the Benjamini–Hochberg procedure was applied to control the false discovery rate (FDR) [31].

## Results

### Socio-demographic and biochemical characteristics of MASLD patients compared to healthy controls

Table 3 illustrates the socio-demographic and biochemical characteristics of the study groups. There was no significant difference in age between MASLD patients (42 ± 6.64 years) and controls (39 ± 8.98 years), and females were predominant in both groups (MASLD, 66.7% vs. control 60%). There was no statistically significant difference in serum Thyroid-Stimulating Hormone (TSH) levels between the MASLD and control groups.

The assessment of lipid profile revealed that the MASLD patients exhibited significantly higher serum triglycerides (169 ± 61.839 vs. 100 ± 37.15 mg/dl, *p* < 0.001) and total cholesterol levels (212 ± 32.019 vs. 199 ± 23.416 mg/dl, *p* < 0.05) compared to controls. Furthermore, liver enzymes were elevated in MASLD, with ALT (41.5 ± 17.863 vs. 29 ± 19.538 IU/L, *p* < 0.01) and AST (43.5 ± 17.28 vs. 22 ± 15.4682 IU/L, *p* < 0.001) both showing significant differences. Additionally, the serum direct bilirubin was significantly higher in MASLD patients, compared to the controls (0.7 ± 0.314 vs. 0.2 ± 0.168 mg/dl, respectively, *p* < 0.001). In contrast, serum albumin and total bilirubin levels did not differ significantly between groups. Renal function parameters, including serum creatinine and uric acid, showed no significant differences between the two groups. MASLD patients demonstrated a distinct biochemical profile characterized by dyslipidemia, elevated transaminases, and increased direct bilirubin.

**Table 3:** Socio-demographic characteristics for control and MASLD patients

Variable	Control group (*N* = 30)	MASLD group (*N* = 30)
**Age**	39 ± 8.98	42 ± 6.64
**Sex**		
**Male**	12 (40%)	10 (33.3%)
**Female**	18 (60%)	20 (66.7%)
**Serum TSH (mU/L)**	0.5 ± 11.305	4.45 ± 15.063
**Complete blood count**		
**Total leukocytic count**	7.5 ± 2.302	7.4 ± 2.562
**Hemoglobin level (g/dl)**	12.2 ± 1.34	11.9 ± 1.024
**Serum lipid profile**		
**Serum total cholesterol (mg/dl)**	199 ± 23.416	212 ± 32.019
**Serum triglycerides (mg/dl)**	100 ± 37.15	169 ± 61.839
**Liver Function test**		
**ALT (IU/L)**	29 ± 19.538	41.5 ± 17.863
**AST (IU/L)**	22 ± 15.4682	43.5 ± 17.28
**Serum albumin (g/dl)**	4.1 ± 0.55	4 ± 0.526
**Serum direct bilirubin (mg/dl)**	0.2 ± 0.168	0.7 ± 0.314
**Serum total bilirubin (mg/dl)**	1 ± 0.206	1 ± 0.332
**INR**	1 ± 0.0069	1.035 ± 0.135
**Renal Function test**		
**Serum creatinine (mg/dl)**	1 ± 0.1818	1 ± 0.176
**Serum uric acid (mg/dl)**	6.5 ± 1.867	5.95 ± 1.76
**FIB4**	0.808	1.19

*Data presented as Mean ± SD unless otherwise stated are presented as %. ALT: Alanine aminotransferase, AST: Aspartate aminotransferase, INR: International normalized ratio, FIB-4: Fibrosis-4 index, MASLD: Metabolic dysfunction-associated steatotic liver disease

### Comparison of biomarkers and gut microbiota between MASLD and control groups

A comparative analysis between MASLD patients and healthy individuals revealed a significant difference in several serum biomarkers and gut microbiota compositions, as illustrated in Table 4; among these biomarkers is TGF-β. Our study showed that the gene expression level of TGF-β was significantly elevated in MASLD patients (median: 163.14; IQR: 163.14–218.27) compared to controls (median: 1.97; IQR: 1.97–29.04), with a highly significant difference (*p* < 0.001). Similarly, the serum level of MDA and gene expression level of STAT3 were higher in the MASLD group, compared to the healthy individuals (MDA: 0.72 vs. 0.311, *p* = 0.002; STAT3: 37.27 vs. 13.17, *p* = 0.048). The FIB-4 index was also notably increased in MASLD patients (median: 1.19) compared to controls (median: 0.808; *p* = 0.017), indicating greater liver fibrosis severity. In contrast, NRF2 gene expression levels were significantly lower in the MASLD group (median: 0.71), compared to controls (median: 53.44; *p* = 0.008), reflecting impaired antioxidant defense mechanisms. Although SOD levels trended lower in MASLD patients, the difference did not reach statistical significance (*p* = 0.072).

Furthermore, our findings revealed a significant dysbiosis between the MASLD and control participants regarding gut microbiota composition. MASLD patients had a markedly higher abundance of *Bacteroides* (median: 0.68) compared to controls (median: 0.18; *p* < 0.001), and significantly reduced levels of *Bifidobacterium* (median: 0.0001 vs. 0.000307; *p* = 0.005). No statistically significant differences were observed in the gene expression levels of *Providencia*, *Porphyromonas*, *Pseudomonas aeruginosa*, *Enterococcus faecium*, *Clostridium*, or *Fusobacterium* between the two groups (*p* > 0.05 for all). These findings highlighted a specific pattern of biomarker alteration and gut microbiome dysbiosis associated with MASLD, suggesting their potential involvement in the disease’s development and progression.

Interestingly, after the brunner-munzel test (BMT) and Benjamini–Hochberg FDR correction, where both tests confirmed the significance differences in TGF-β, MDA, NRF2, FIB-4, *Bacteroides*, and *Bifidobacterium* remained statistically significant, whereas STAT3 did not retain significance.

**Table 4:** Biomarkers and microbiome levels in MASLD vs. control

Measurement	Control		MASLD		Sig. (MWT)	Sig. (BMT)	Benjamini–Hochberg FDR -adjusted *p*- value
	Median	Q1-Q3	Median	Q1-Q3			
TGF-β	1.97	1.97–29.04	163.14	163.14–218.27	**< 0.001** ^**^	<. 001**	**0.0028** ^*^
SOD	97.78	0.24–97.79	92.26	−208.02–97.78	0.072	0.097	0.1260
MDA	0.311	0.311–0.722	0.72	0.31–1.47	**0.002** ^*^	0.001**	**0.0093** ^*^
Stat3	13.17	3.012–37.27	37.27	10.33–37.27	**0.048** ^*^	0.051	0.0960
NRF2	53.44	0.97–54.7	0.71	0.72–53.44	**0.008** ^*^	0.007**	**0.0224** ^*^
FIB4	0.808	0.59–1.089	1.19	0.89–1.65	**0.017** ^*^	0.017**	**0.0397** ^*^
*Bifidobacterium*	0.000307	0.0001–0.0003	0.0001	0.0001 − 0.0003	**0.005** ^*^	0.004**	**0.0175** ^*^
*Bacteroides*	0.18	0.012–0.485	0.68	0.3–1.06	**< 0.001** ^**^	< 0.001**	**0.0028** ^*^
*Providencia*	0.506	0.147–1.141	0.071	0.000–1.14	0.130	0.135	0.2022
*Porphyromonas*	3.251	2.62–4.406	4.28	1.99–6.06	0.135	0.148	0.2025
*Pseudomonas aeruginosa*	0.0000025	0.0000011–0.000061	0.000061	0.0000011–0.000061	0.335	0.340	0.4690
*Enterococcus faecium*	1.844	0.69–3.49	2.4	0.89–3.64	0.505	0.510	0.5383
*Clostridium*	1.097	0.36–2.81	1.68	0.43–2.45	0.801	0.805	0.8010
*Fusobacterium*	0.181	0.005–0.74	0.39	0.005 − 0.62	0.452	0.461	0.5273

*Q1-Q3 = 25th – 75th percentile. Sig. =statistical significance (significant at p-value < 0.05^*^ and < 0.001^**^). The test of significance is Mann-Whitney U-test. TGF-β = transforming growth factor beta, SOD = superoxide dismutase, MDA = malondialdehyde, STAT3 = signal transducer and activator of transcription 3, NRF2 = nuclear factor erythroid 2-related factor 2, FIB-4 = fibrosis-4 (Index for Liver Fibrosis). FDR: Benjamini–Hochberg false discovery rate correction. Bold values represent a statistical significance at p-value <.05* and <.001** and best performance of different parameters

### Diagnostic performance evaluation of circulating biomarkers for MASLD

**Fig. 1:**
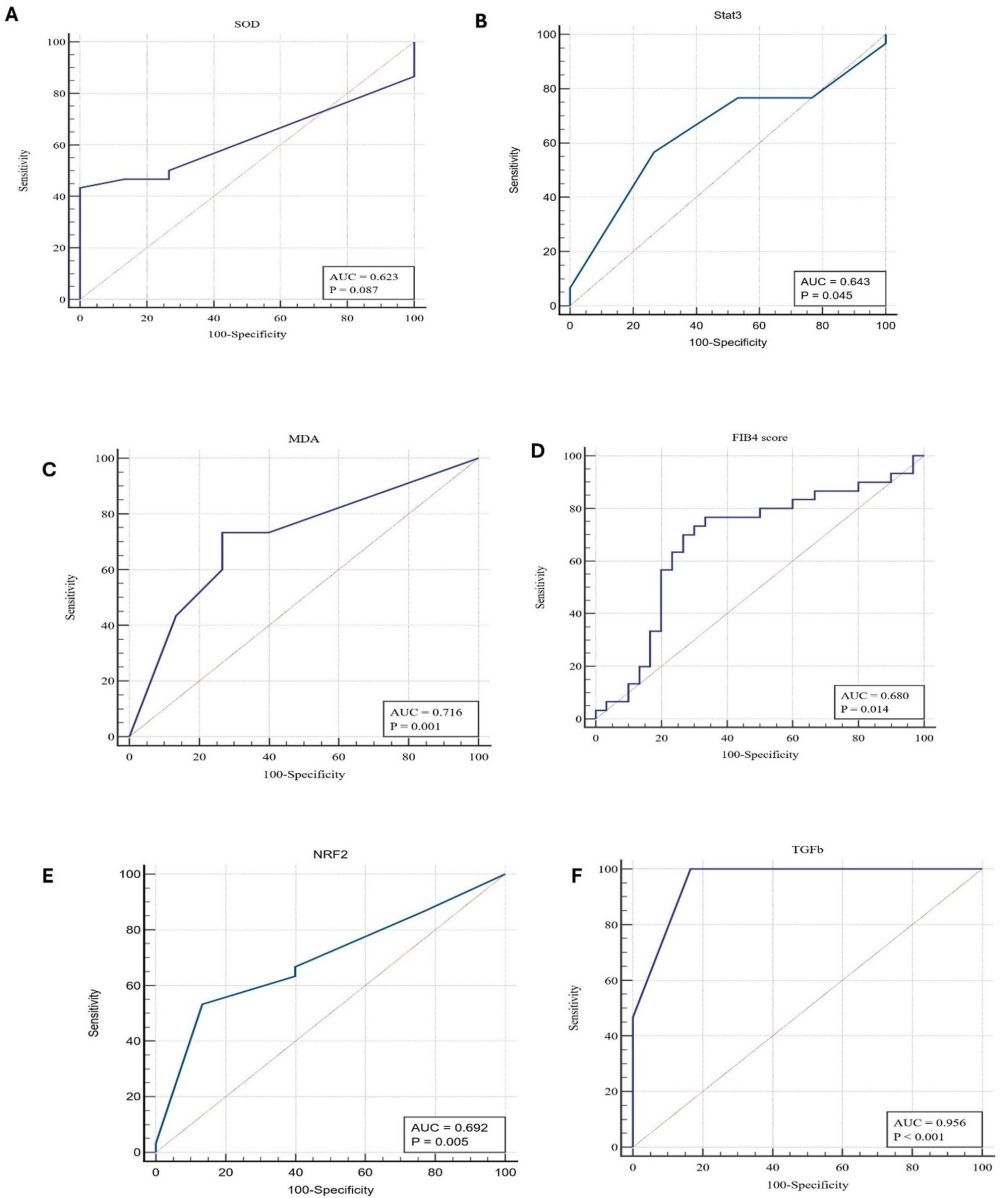
Receiver operating characteristic (ROC) curves illustrating the diagnostic performance of different biomarkers (A) SOD, (B) STAT3, (C) MDA, (D) FIB-4 score, (E) NRF2, and (F) TGF-β in discriminating MASLD. TGF-β = Transforming Growth Factor Beta, SOD = Superoxide dismutase, MDA = Malondialdehyde, STAT3 = Signal Transducer and Activator of Transcription 3, NRF2 = nuclear factor erythroid 2-related factor 2, FIB-4 = Fibrosis-4 (Index for Liver Fibrosis)

The diagnostic performance of various biomarkers in identifying MASLD was assessed using receiver operating characteristic (ROC) curve analysis. To mitigate the risk of “overoptimistic inflated results” often associated with standard metrics, our findings were validated using the Matthews Correlation Coefficient (MCC). As established by Chicco & Jurman, 2020 [32], MCC is a more “informative and truthful” metric than Accuracy or F1 score because it only produces a high score if the model performs well across all four categories of the confusion matrix (True Positives, True Negatives, False Positives, and False Negatives), Table 5, and Fig. 1.

The TGF-β exhibited the highest diagnostic accuracy, with an AUC of 0.956 (95% CI: 0.869–0.992, *p* < 0.0001). At a cutoff value of > 92.41, TGF-β showed 100% sensitivity, 83.33% specificity, 85.71% PPV, 100% NPV, and % overall accuracy of 91.66%. The F1 score and Matthews Correlation Coefficient (MCC) for TGF-β were 92.3% and 84.51%, respectively, indicating strong predictive performance.

Among oxidative stress-related markers, MDA showed a relatively good diagnostic ability with an AUC of 0.716 (95% CI: 0.584–0.825, *p* = 0.0008) at a cutoff > 0.349. It achieved 73.33% sensitivity, 73.33% specificity, and 66.67% accuracy, with a moderate F1 score of 68.75% and MCC of 33.63%. The fibrosis marker, FIB-4 index, showed moderate performance with an AUC of 0.680 (95% CI: 0.547–0.795, *p* = 0.0141) at a cutoff > 0.91. It yielded 76.67% sensitivity and 66.67% specificity, resulting in % overall accuracy of 70%. NRF2 and STAT3 also demonstrated good diagnostic capabilities with AUCs of 0.692 and 0.643, respectively. NRF2 (cutoff ≤ 0.72) achieved high specificity (86.67%) but lower sensitivity (53.33%), whereas STAT3 (cutoff > 32.72) showed more balanced but suboptimal sensitivity (56.67%) and specificity (73.33%). Furthermore, although SOD had perfect specificity (100%) at a cutoff ≤ −104.7, its sensitivity was low (43.33%), resulting in an AUC of 0.623 (95% CI: 0.489–0.745, *p* = 0.087), suggesting limited diagnostic value. Overall, TGF-β emerged as the most promising biomarker for MASLD diagnosis, demonstrating excellent sensitivity, specificity, and overall diagnostic performance.

**Table 5:** Diagnostic performance of biomarkers in diagnosing of MASLD:

Biomarker	Cutoff	AUC	95% CI		Sig.	SN %	SP %	PPV %	NPV %	ACC %	F1 score %	MCC %
			Lower	Upper								
FIB4	> 0.91	0.680	0.547	0.795	0.0141*	76.67	66.67	68.75	71.43	70	70.97	40.19
TGF-β	> 92.41	0.956	0.869	0.992	< 0.0001**	**100**	83.33	85.71	**100**	91.66	92.3	84.51
NRF2	≤ 0.72	0.692	0.56	0.805	0.0045**	53.33	86.67	80	65	70	64	42.43
STAT3	> 32.72	0.643	0.509	0.763	0.0451*	56.67	73.33	68	62.86	65	61.82	30.43
SOD	≤ −104.7	0.623	0.489	0.745	0.087	43.33	**100**	**100**	63.83	71.67	60.46	52.59
MDA	> 0.349	0.716	0.584	0.825	0.0008**	73.33	73.33	64.71	69.23	66.67	68.75	33.63

*TGF-β = Transforming Growth Factor Beta, SOD = Superoxide dismutase, MDA = Malondialdehyde, STAT3 = Signal Transducer and Activator of Transcription 3, NRF2 = nuclear factor erythroid 2-related factor 2, FIB-4 = Fibrosis-4 (Index for Liver Fibrosis). Bold values indicate the best performance and statistical significance at p-value <.05* and <.001**)

### The evaluation of gut microbiomes as potential non-invasive biomarkers for MASLD

**Fig. 2:**
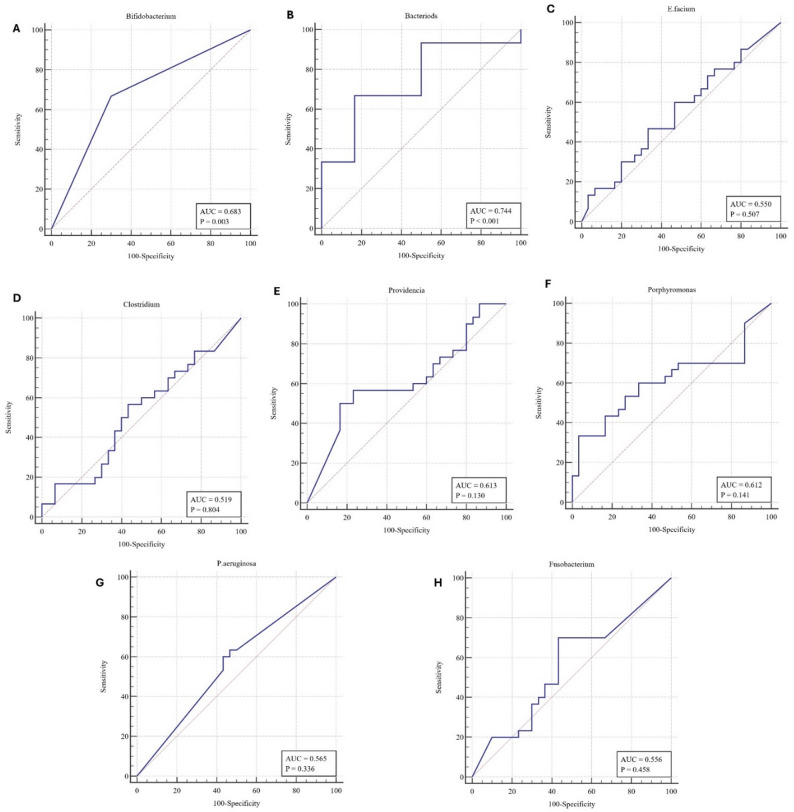
Receiver Operating Characteristic (ROC) Curves showing the diagnostic performance of classification models for eight different bacterial species: (**A**) *Bifidobacterium*, (**B**) *Bacteroides*, (**C**) *E. faecium*, (**D**) *Clostridium*, (**E**) *Providencia*, (**F**) *Porphyromonas*, (**G**) *P. aeruginosa*, and (**H**) *Fusobacterium*

The diagnostic performance of different gut microbiomes in distinguishing MASLD patients from healthy controls was assessed using ROC curve analysis, as summarized in Table 6 and Fig. 2. *Bacteroides* demonstrated the highest diagnostic accuracy among the analyzed bacterial taxa, with an AUC of 0.744 (95% CI: 0.615–0.848, *p* = 0.0002), a sensitivity of 66.67%, and a specificity of 83.33%. It also showed the highest Matthews correlation coefficient (MCC = 47.62%), indicating a relatively strong discriminatory ability. Furthermore, *Bifidobacterium* showed the second diagnostic performance among the assessed bacterial species with an AUC of 0.683 (95% CI: 0.550–0.797, *p* = 0.0027), sensitivity and specificity of 66.67% and 70.00%, respectively, and a balanced performance with an MCC of 36.68%. On the other hand, although *Providencia* and *Porphyromonas* exhibited relatively high specificity (83.33% and 96.67%, respectively), their sensitivity was low (50.00% and 33.33%), and their AUCs did not reach statistical significance (*p* > 0.05).

Conversely, other species showed limited diagnostic utility, including *P. aeruginosa*, *E. faecium*, *Clostridium* Sp., and *Fusobacterium*, with AUCs values, 0.565, 0.55, 0.519, and 0.556, respectively, with non-significant p-values. The overall performance metrics, such as accuracy, F1 score, and MCC, were consistently lower for these bacteria, indicating limited potential as standalone diagnostic markers for MASLD. Collectively, our findings revealed that specific alterations in gut microbiota, particularly increased levels of *Bacteroides* and reduced levels of *Bifidobacterium*, may serve as promising non-invasive biomarkers for diagnosing MASLD.

**Table 6:** Diagnostic performance of Gut Microbiota in diagnosing of MASLD:

Biomarker	Cutoff	AUC	95% CI		Sig.	SN %	SP %	PPV %	NPV %	ACC %	F1 score %	MCC %
			Lower	Upper								
*Bacteroides*	> 0.502	0.744	0.615	0.848	**0.0002** ^**^	66.67	83.33	79.16	69.44	73.33	70.37	47.62
*Bifidobacterium*	≤ 0.0001	0.683	0.550	0.797	**0.0027** ^*^	66.67	70.00	68.96	67.74	68.33	67.79	36.68
*Providencia*	≤ 0.035	0.613	0.478	0.736	0.139	50.00	83.33	75	62.5	66.67	60	35.35
*Porphyromonas gingivalis*	> 5.96	0.612	0.478	0.735	0.141	33.33	96.67	83.33	58.33	63.33	47.62	33.33
*P. aeruginosa*	> 0.000004	0.565	0.431	0.693	0.336	63.33	53.33	57.57	59.26	58.33	60.32	16.75
*E. faecium*	> 2.037	0.55	0.416	0.679	0.5	60.00	53.33	56.25	57.14	56.67	58.06	13.36
*Clostridium*	> 1.2	0.519	0.38	0.65	0.8	56.67	56.67	56.67	56.67	56.67	56.67	13.33
*Fusobacterium*	> 0.2021	0.556	0.422	0.684	0.458	70.00	56.67	61.76	65.38	63.33	65.63	26.91

*AUC = area under the ROC curve, 95%CI = 95% confidence interval, Sig = significance, SN = sensitivity, SP = Specificity, PPV = positive predictive value, NPV = negative predictive value, ACC = accuracy, MCC = Matthews correlation coefficient. Bold values represent the best performance and statistical significance (significant at p-value <.05* and <.001**)

### Correlation between serum lipid profiles, gut microbiomes, and biomarker levels in MASLD

**Fig. 3:**
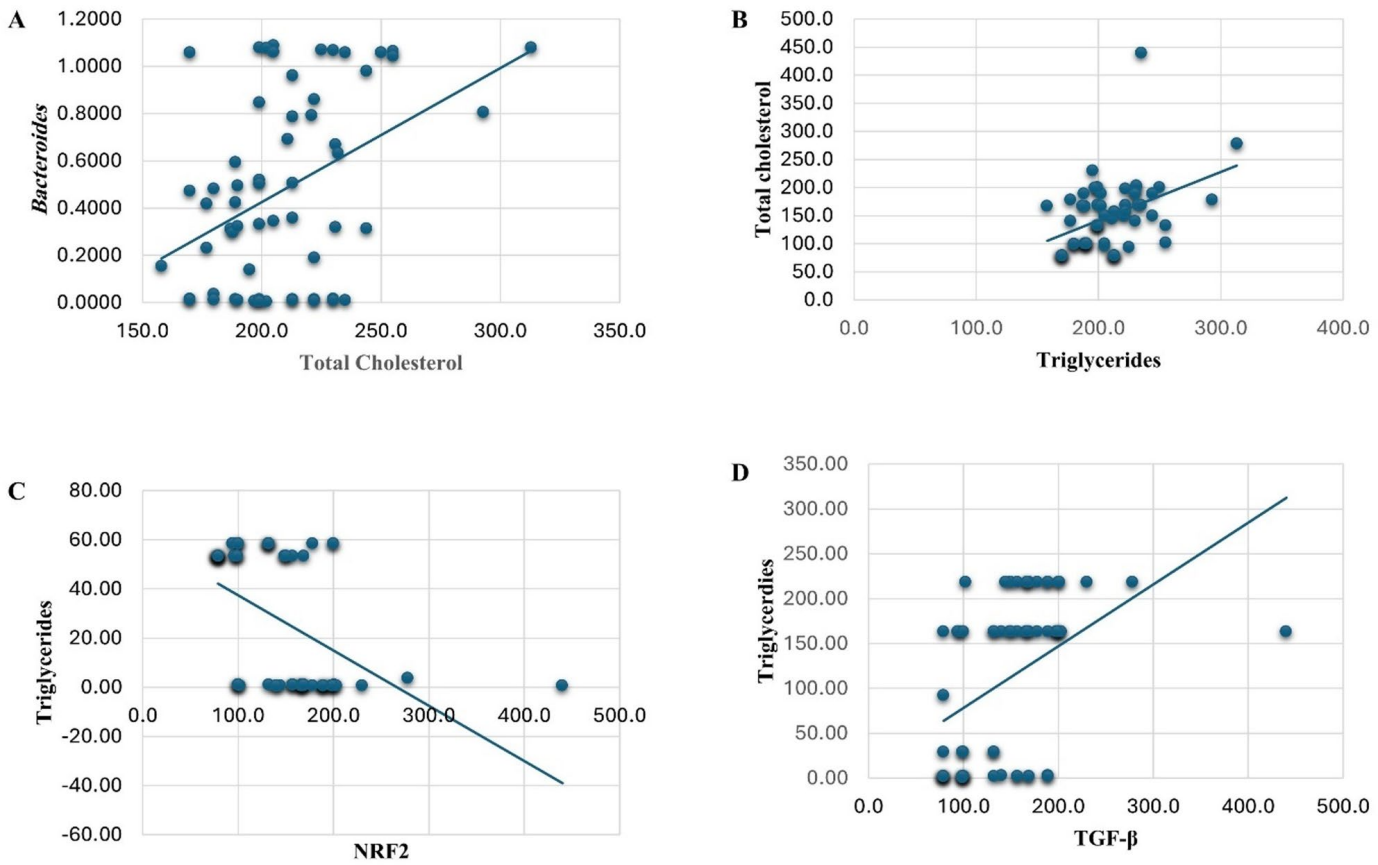
Correlation analysis between clinical parameters. Scatter plots showing correlations with linear regression lines. (**A**) Positive correlation between *Bacteroides* abundance and total cholesterol levels. (**B**) Positive correlation between total cholesterol and triglycerides. (**C**) Negative correlation between triglycerides and NRF2 expression. (**D**) Positive correlation between TGF-β and triglycerides. TGF-β = Transforming Growth Factor Beta and NRF2 = nuclear factor erythroid 2-related factor 2

Spearman’s correlation analysis was conducted to assess the relationships between serum lipid profiles, total cholesterol (TC) and triglycerides (TG), gut microbiota composition, and selected serum biomarkers, as illustrated in Table 7 and Fig. 3.

Serum TG showed a significant positive correlation with TC (rs = 0.398, *p* = 0.002). Among the gut microbiomes, the abundance of *Bifidobacterium* was inversely correlated with TC (rs = − 0.315, *p* = 0.014), while the abundance of *Bacteroides* demonstrated a positive correlation (rs = 0.330, *p* = 0.010). No significant correlations were observed between TC and other assessed bacterial species. On the other hand, for TG, TGF-β exhibited a strong positive correlation (rs = 0.528, *p* < 0.001), whereas NRF2 showed a strong inverse correlation (rs = − 0.503, *p* < 0.001). None of the gut microbiomes showed statistically significant correlations with TG levels, although *Porphyromonas* displayed a borderline positive association (rs = 0.250, *p* = 0.054). Conversely, no significant correlations were observed between lipid profiles and other biomarkers, including FIB-4, STAT3, SOD, and MDA. These findings suggest that *Bifidobacterium*, *Bacteroides*, TGF-β and NRF2 may be associated with cholesterol regulation, in MASLD patients.

After Benjamini–Hochberg FDR correction, significant correlations persisted between serum triglycerides and total cholesterol (adjusted *p* = 0.010), as well as between triglycerides and both TGF-β (adjusted *p* = 0.003) and NRF2 (adjusted *p* = 0.003). Additionally, *Bacteroides* and *Bifidobacterium* remained significantly correlated with total cholesterol.

**Table 7:** Correlation of TC and TG with biomarkers and microbiome:

Marker	Serum total cholesterol (TC)			Serum triglycerides (TG)		
	Rs	Sig.	Corrected *p*-value (FDR)	Rs	Sig.	Corrected *p*-value (FDR)
Serum triglycerides (TG)	0.398**	0.002**	0.030*	1.000		
*E. facium*	− 0.045	0.732	0.891	0.189	0.148	0.443
*P. aeruginosa*	0.038	0.772	0.891	0.033	0.805	0.805
*Fusobacterium*	0.056	0.671	0.891	0.081	0.539	0.580
*Porphyromonas*	0.013	0.919	0.919	0.250	**0.054**	**0.252**
*Clostridium*	0.149	0.257	0.482	0.111	0.400	0.526
*Providencia*	− 0.040	0.763	0.891	−0.108	0.413	0.526
*Bifidobacterium*	−0.315*	0.014*	0.070	−0.119	0.366	0.526
*Bacteroides*	0.330*	0.010*	0.070	0.137	0.298	0.526
FIB4	−0.151	0.248	0.482	−0.174	0.184	0.443
TGF-β	0.192	0.141	0.423	0.528*	< 0 0.001**	0.007*
NRF2	−0.199	0.127	0.423	−0.503*	< 0.001**	0.007*
STAT3	−0.022	0.867	0.919	0.122	0.354	0.526
SOD	−0.163	0.213	0.482	−0.095	0.471	0.550
MDA	0.067	0.610	0.891	0.171	0.190	0.443

*r_s_ = Correlation coefficient, Sig. =statistical significance (significant at p-value < 0.05^*^ and < 0.001^**^). TGF-β = Transforming Growth Factor-Beta, SOD = Superoxide dismutase, MDA = Malondialdehyde, STAT3 = Signal Transducer and Activator of Transcription 3, NRF2 = nuclear factor erythroid 2-related factor 2, FIB-4 = Fibrosis-4 (Index for Liver Fibrosis). Bold values represent best performance and statistical significance

## Discussion

In this case–control study of Egyptian adults, we identified distinct gut microbiome dysbiosis and biochemical alterations associated with metabolic dysfunction–associated steatotic liver disease (MASLD). Our findings demonstrated that the gene expression of TGF-β was significantly elevated in MASLD patients and provided an excellent diagnostic performance, with an AUC of 0.956, outperforming conventional fibrosis indices such as FIB-4. We also observed characteristic microbial dysbiosis, especially the enrichment of *Bacteroides* and depletion of *Bifidobacterium*, which significantly correlated with lipid profiles and disease-related biochemical markers. These findings provide novel evidence that integrating circulating biomarkers with targeted microbial profiling could improve non-invasive MASLD diagnosis and shed light on the gut–liver axis in disease pathogenesis.

Recent studies have increasingly highlighted the gut–liver axis as a chief cause of MASLD development and progression. In this context, the gut microbiomes contribute to influencing the liver’s response to metabolic stress, inflammation, and fibrosis by exerting their metabolic and immune-regulatory effects [33]. Alterations in gut microbiome composition, known as dysbiosis, are associated with the pathogenesis of MASLD, with significant impacts on liver function and the overall metabolic role [34]. Similarly, our findings revealed that the MASLD group displayed a substantial elevation in the abundance of *Bacteroides* alongside a decline in *Bifidobacterium.* This dysbiosis altered the metabolic role of the gut microbiome, potentially modifying the host’s exposure to factors associated with MASLD pathogenesis. This is the first study to investigate gut dysbiosis in MSALD patients from the Egyptian continent. In non-alcoholic steatohepatitis, the abundance of *Bacteroides* was significantly increased in European patients [35]. A previous study also showed that *Bacteroides*-driven alterations in the intestinal microbiota increased the lipopolysaccharide levels and impaired lipid and glucose metabolism, thus aggravating NAFLD in mice [36]. In contrast to *Bifidobacterium*, a previous study illustrated that *Bifidobacterium* was found to be the top depleted bacterial taxa in NAFLD-associated hepatocellular carcinoma in mice [37]. Another study exhibited that *Bifidobacterium* alleviated NAFLD by regulating the de novo synthesis of lipid and inhibiting the inflammation [38]. Additionally, researchers have demonstrated that *Bifidobacterium* levels are decreased in different conditions, including major depressive disorder and diabetes [39, 40]. In our study, while neither *Bacteroides* nor *Bifidobacterium* individually achieves perfect discrimination, their statistically significant AUCs, acceptable sensitivity and specificity, and balanced predictive metrics indicate they are informative microbial signals for MASLD classification. These results are consistent with the broader literature on microbiome biomarkers where individual taxa often yield moderate AUCs but contribute meaningfully to ensemble or multimodal diagnostic models [41, 42].

Published studies pointed to several mechanisms that may underline the relation between dysbiosis of gut microbiota and the promotion of MASLD. *Bacteroides* abundance has been positively correlated with fecal levels of deoxycholic acid, choline, D-pinitol, raffinose, and stachyose. In contrast, fecal *Bacteroides* levels have shown negative correlations with amino acids and fecal short-chain fatty acids (SCFAs) [43]. Most of these compounds contribute to the pathogenesis of MASLD. For example, deoxycholic acid induced MASLD development in mice by disrupting lipid homeostasis and modulating inflammation and fibrosis [44]. Furthermore, SCFAs help regulate steatosis of hepatic tissues and inflammation *via* the gut–liver axis, playing a key role in mitigating NAFLD and MASLD [45]. Hence, *Bacteroides*-associated increases in deoxycholic acid and decreased SCFAs might be unfavorable for MASLD development. Conversely, *Bifidobacterium* species contribute to SCFA production, intestinal barrier integrity, and anti-inflammatory responses [46]. The inverse correlation between *Bifidobacterium* and total cholesterol levels in our cohort suggests a possible lipid-lowering role, consistent with prior experimental evidence showing probiotic supplementation reduces hyperlipidemia and hepatic fat accumulation [47]. These microbiome alterations suggest a mechanistic link between dysbiosis, lipid metabolism, and MASLD progression.

Fusobacterium is a Gram-negative bacterium that favors anaerobic conditions. They produce short-chain fatty acids (SCFAs). It has been linked to stimulating the inflammation and inducing multiple cytokine production, contributing to chronic inflammation as well as carcinogenesis [48]. Furthermore, previous investigations reported that *Fusobacterium* and *Escherichia–Shigella* served as independent causes related to fibrosis severity in MASLD patients. Notably, *Fusobacterium* abundance positively correlated with Magnetic Resonance Elastography values and was found to be more prevalent in steatohepatitis patients compared to those with simple steatosis, indicating its potential role in MASLD progression [49, 50]. In the same line, our findings revealed an elevation in the abundance of *Fusobacterium* in MASLD patients, compared to the healthy participants; however, this elevation was still non-significant.

Similarly, *Clostridium* species is a chief group of commensal gut bacteria, which contributes to maintaining intestinal homeostasis. Dietary patterns can influence their abundance, and *Clostridium* spp. support energy supply to intestinal epithelial cells, reinforce the gut barrier, and modulate immune responses. Their metabolites, including secondary bile acids (BAs), short-chain fatty acids, and indole propionic acids, have been linked to metabolic disorders and obesity. Previous studies reported significant disturbances in bile acid–related bacteria, particularly *Clostridium* spp., in gut microbiota. In advanced stages of NAFLD with severe liver injury, elevated levels of *Clostridium* spp., were accompanied by increased secondary bile acids, notably lithocholic acid (LCA) and its derivatives [48]. LCA exhibits cytotoxic activities that induce membrane damage and are strongly associated with advanced fibrosis and hepatocellular damage [51].

Additionally, *E. faecium* may have a role in NAFLD development through the production of tyramine, the bioactive metabolite, which is thought to interact with PPAR-γ, a vital ligand-activated receptor in the PPAR signaling pathway, thereby disrupting lipid metabolism and promoting inflammatory damage and liver fibrosis [52]. To the contrary of these findings, *Enterococcus* promotes macrophage activation via the NF-κB signaling pathway, elevates profibrotic mediators like TGF-β and MCP-1 in liver tissue, and accelerates fibrogenesis marked by increased α-SMA and collagen I expression [53]. Interestingly, in a contrasting context, certain formulations containing *E. faecium*, notably a probiotic cocktail with *Bacillus subtilis*, have shown protective effects in NAFLD models by reducing inflammatory cytokines (IL-1β, IL-6, TNF-α) and downregulating hepatic TLR4–NF-κB signaling [54]. Adding to the previous study, *E. faecium* appears to exert hepatoprotective effects via its extracellular vesicles (EfmEVs) that reduce ALT and AST levels, and fortify the antioxidant defenses [55].

As a major pathogen frequently detected in periodontitis, *Porphyromonas gingivalis* (*P. gingivalis*) has been associated with an increased risk of insulin resistance, metabolic syndrome, NAFLD, and NASH [56]. Cell surface components of *P. gingivalis*, including lipopolysaccharides, fimbriae, and outer membrane protein A, serve as its major pathogenic factors [57, 58]. *P. gingivalis* is believed to induce NAFLD development through multiple mechanisms, including inflammation, endotoxemia, oxidative stress, macrophage polarization, metabolic remodeling, and alterations in the gut microbiome [59, 60].

Gut dysbiosis, characterized by an imbalance in the microbiome of the gut, causes a significant increase in reactive oxygen species (ROS) production, and a decrease in the antioxidant enzymes which in turn promotes the inflammatory process, immune activation, DNA damage, and epigenetic modifications of key metabolic genes [61]. MASLD is primarily characterized by the buildup of triglycerides in liver cells [62]. Hepatocyte lipid accumulation induces lipotoxicity, subsequently activating oxidative stress pathways, initiating cell death cascades that drive progression from hepatic steatosis to inflammation and fibrosis [63]. Our biochemical profiling revealed increased oxidative stress and pro-fibrogenic signaling in MASLD, as evidenced by elevated MDA and TGF-β alongside decreased NRF2 expression and SOD levels. TGF-β plays a pivotal role in fibrogenesis. In fibrotic diseases, TGF-β expression and activation are increased, driving changes in fibroblast phenotypes and function, stimulating their trans-differentiation into myofibroblasts, and enhancing extracellular matrix preservation [64]. In our study, we acknowledge that TGF-β is a nonspecific cytokine frequently elevated in diverse systemic inflammatory and fibrotic conditions. To ensure the diagnostic relevance of circulating TGF-β specifically within the context of MASLD, several methodological measures were implemented. First, our study utilized strict exclusion criteria, omitting individuals with other chronic liver diseases, malignancy, inflammatory bowel disease, and recent antibiotic or probiotic use, all of which are potential confounding systemic sources of TGF-β. Furthermore, our results demonstrated that TGF-β expression was not only significantly elevated in MASLD patients compared to healthy controls (median: 163.14 vs. 1.97, *p* < 0.001) but also exhibited a strong positive correlation with serum triglycerides (*r* = 0.528, *p* < 0.001), a relationship that remained statistically significant after Benjamini–Hochberg False Discovery Rate correction (*p* = 0.003). This specific metabolic correlation, alongside its excellent diagnostic performance within our cohort (AUC = 0.956, 100% sensitivity), suggests that the observed TGF-β elevations are closely linked to the metabolic and hepatic alterations characteristic of MASLD rather than generalized systemic inflammation.

Meanwhile, NRF2 is a key transcription factor supporting electrophile detoxification and sustaining metabolic balance, regulating bile acid enterohepatic circulation and bile secretion [65]. The observed changes in NRF2 levels in MASLD patients likely reflect an impaired antioxidant defense and metabolic regulatory capacity. This disruption is further supported by the elevated MDA levels and decreased SOD levels detected in our MASLD cohort. MDA, a lipid peroxidation byproduct, is a reliable marker of oxidative damage to cellular membranes, reflecting ROS generation [66]. As part of the cellular defense against oxidative damage, superoxide dismutase catalyzes the conversion of superoxide radical anions to hydrogen peroxide [67]. In parallel, our findings of elevated STAT3 expression point to the activation of inflammatory and pro-fibrotic signaling cascades. STAT3 is a key contributor to the pathogenesis of liver diseases [68]. It is a cytoplasmic transcription factor within the Janus kinase (JAK)–signal transducer and activator of transcription (STAT) pathway, which plays a central role in mediating liver injury. Through this pathway, various cytokines and growth factors exert critical regulatory effects in mammals, driving processes such as hepatic tissue remodeling, extracellular matrix deposition, and chronic inflammation [69]. This dysregulation may contribute to hepatic oxidative stress, lipid accumulation, and inflammation, thereby exacerbating disease progression.

Our findings identify a multi-dimensional biomarker signature with strong potential for non-invasive MASLD diagnosis. TGF-β demonstrated excellent discriminative power, reflecting its central role in fibrogenesis, while the FIB-4 index provided a simple, clinically accessible measure of fibrosis severity. The combination of elevated MDA and reduced NRF2 captured the oxidative stress–antioxidant imbalance that drives disease progression, and increased STAT3 levels highlighted concurrent inflammatory and pro-fibrotic activity. These molecular and biochemical markers were significantly complemented by distinct microbiome alterations, *Bacteroides* enrichment, and *Bifidobacterium* depletion, linking systemic pathology to gut–liver axis disruption. A previous study illustrated that the gut TGF-β expression is markedly reduced in germ-free mice [70]. Multiple Clostridium species have been reported to generate short-chain fatty acids such as propionate, acetate, and butyrate that can enhance the production of TGF-β in colonic epithelial cells [71]. Furthermore, Clostridium species not only enhance TGF-β secretion by colonic epithelial cells but also upregulate metalloproteinase expression on their surface, thereby creating a substantial source of bioactive TGF-β in the colon [72]. Microbiota-derived products have also been shown to regulate TGF-β generation by lamina propria dendritic cells, including adenosine triphosphate, which enhances the expression of TGF-β in a CD70-high subset of dendritic cells in the small intestine. Like the action of short-chain fatty acids on intestinal epithelial cells, microbiota-derived adenosine triphosphate can directly stimulate TGF-β production in intestinal epithelial cells and indirectly elevate active TGF-β levels by inducing integrin alpha-v beta-8 expression on the surface of dendritic cells [73]. This integrated biomarker profile could enhance diagnostic accuracy, reduce reliance on liver biopsy, and enable earlier, targeted intervention. Validation in larger cohorts is warranted to confirm its clinical utility.

Our results revealed a distinct correlation between gut microbiota and altered serum biomarkers in MASLD. *Bifidobacterium* abundance inversely correlated with total cholesterol, supporting its lipid-lowering and anti-inflammatory roles. Our findings agreed with another research that found probiotic supplements enriched with *Bifidobacterium* attenuate NAFLD by regulating β-oxidation, lipogenesis, and inflammatory signaling [74]. On the other hand, *Bacteroides* abundance is positively *correlated* with cholesterol, which is consistent with its association with unfavorable bile acid profiles and lipid accumulation [75]. Another study revealed that *Bacteroides* abundance positively correlated with total cholesterol in a high-fat diet mouse model [76]. Additionally, TGF-β showed a strong positive correlation with triglycerides, linking hypertriglyceridemia to enhanced fibrosis signaling [77], while NRF2 correlated negatively with triglycerides, reflecting reduced antioxidant defense in lipid-rich conditions [78]. These patterns highlight the gut–liver–lipid axis as a key driver of MASLD, suggesting that microbiome modulation and oxidative stress regulation could simultaneously improve lipid metabolism and attenuate disease progression. Strategies such as *Bifidobacterium*-enriched probiotics, prebiotics, or dietary fiber supplementation may help restore gut–liver homeostasis. Moreover, interventions aimed at enhancing NRF2 activation or dampening TGF-β/STAT3 signaling could mitigate oxidative and inflammatory damage. Our findings also underscore the need to integrate microbiome monitoring into MASLD management to guide personalized treatment approaches.

Despite the promising findings, a recommendation of this study includes future multi-center larger-scale validation studies to generalize these findings. Additionally, gut microbiota profiling in this study was based on qPCR quantification of selected bacterial taxa, which provides targeted, hypothesis-driven insights but does not capture overall microbial diversity or community structure. Therefore, the observed alterations should be interpreted as specific microbial shifts rather than comprehensive gut dysbiosis. Sequencing-based approaches are needed in future studies to confirm and expand these findings. Furthermore, microbial abundance was also assessed using relative quantification normalized to total bacterial load. This approach reflects proportional changes in selected taxa but does not capture absolute microbial counts or global microbiome shifts. Therefore, interpretations should focus on specific taxa rather than overall microbial diversity. Future studies using liver-targeted assessments and sequencing-based or absolute quantification methods are warranted to address these limitations.

## Conclusion

This study highlights the diagnostic potential of integrating circulating biomarkers with gut microbiome signatures in metabolic dysfunction–associated steatotic liver disease (MASLD). Among the evaluated parameters, serum TGF-β exhibited excellent diagnostic accuracy, outperforming conventional indices, while specific microbial alterations, increased *Bacteroides* and decreased *Bifidobacterium*, were significantly associated with lipid dysregulation and disease markers. These findings underscore the pivotal role of the gut–liver axis in MASLD pathogenesis and point toward a combined biomarker–microbiome panel as a promising, non-invasive approach for early detection and risk stratification. Targeting gut dysbiosis and modulating oxidative and fibrotic signaling may represent viable therapeutic avenues. Future large-scale, longitudinal, and multi-omics studies are warranted to validate these signatures and facilitate their translation into routine clinical practice.

## Data Availability

Data are available from the corresponding author on reasonable request.
